# Medicines discarded in household garbage: analysis of a pharmaceutical waste sample in Vienna

**DOI:** 10.1186/2052-3211-7-6

**Published:** 2014-06-11

**Authors:** Sabine Vogler, Christine Leopold, Christel Zuidberg, Claudia Habl

**Affiliations:** 1 WHO Collaborating Centre for Pharmaceutical Pricing and Reimbursement Policies, Health Economics Department, Gesundheit Österreich GmbH/Geschäftsbereich ÖBIG – Austrian Health Institute, Vienna, Austria; 2 WHO Collaborating Centre for Pharmaceutical Policy and Regulation, Division of Pharmacoepidemiology and Clinical Pharmacology, Utrecht Institute for Pharmaceutical Sciences, Utrecht University, Utrecht, The Netherlands

**Keywords:** Austria, Medical waste disposal, Medication adherence, Medicines utilization, Pharmacy services

## Abstract

**Objectives:**

To analyze a sample of pharmaceutical waste drawn from household garbage in Vienna, with the aim to learn whether and which medicines end up unused in normal household waste.

**Methods:**

We obtained a pharmaceutical waste sample from the Vienna Municipal Waste Department. This was drawn by their staff in a representative search in October and November 2009. We did a manual investigation of the sample which contained packs and loose blisters, excluded medical devices and traced loose blisters back to medicines packs. We reported information on the prescription status, origin, therapeutic group, dose form, contents and expiry date. We performed descriptive statistics for the total data set and for sub-groups (e.g. items still containing some of original content).

**Results:**

In total, 152 packs were identified, of which the majority was prescription-only medicines (74%). Cardiovascular medicines accounted for the highest share (24%). 87% of the packs were in oral form. 95% of the packs had not expired. 14.5% of the total data set contained contents but the range of content left in the packs varied. Results on the packs with contents differed from the total: the shares of Over-the Counter medicines (36%), of medicines of the respiratory system (18%) and of the musculo-skeletal system (18%), for dermal use (23%) and of expired medicines (19%) were higher compared to the full data set.

**Conclusions:**

The study showed that some medicines end up unused or partially used in normal household garbage in Vienna. Our results did not confirm speculations about a high percentage of unused medicines improperly discarded. There is room for improved patient information and counseling to enhance medication adherence and a proper discharge of medicines.

## Introduction

Promoting a more rational use of medicines is a major aim of pharmaceutical policy [1-4]. One element of an irrational use of medicines is non-adherence to medication which constitutes a major public health concern all over the world [5-8].

Consequences of non-adherence to medication are multi-fold: Patients’ non-adherence to medicines is expected to substantially impact the effectiveness of the therapy, potentially leading to failures of treatment, side effects and adverse reactions, negative outcomes of morbidity and mortality. This may eventually require more resources (e.g. re-hospitalization, further medication) [9-13]. The under-use and non-use of prescribed, dispensed and/or purchased medicines can result in a substantial economic loss: A study estimated the value of unused prescription medicines for the elderly population above 65 of at least $ 1 billion per year in the United States [14]. For Switzerland, the annual value of unused medicines which end up in household garbage was estimated to amount to CHF 500.- million (more than € 400.- million) [15]. A survey undertaken by the Viennese Sickness Fund reported that medicines worth € 8.1 million which had expired or were simply no longer intended to be taken were estimated to be stored in Viennese households [16]. A recent study estimated a gross annual prescribed medicines wastage of GBP 300 million for the National Health Service in England, which included an estimated GBP 90 million worth of unused prescription medicines that were retained in individuals’ homes at any one time and GBP 110 million returned to community pharmacies over the course of a year [17]. Other studies, even if they could not assess the real value of unused medicines or were based on a small sample, indicated an enormous burden on the pharmaceutical budget caused by unused medicines [18-23].

If medicines are not discarded properly, this may have hazardous consequences to the environment. It may result in polluting the surface and ground water, thus limiting the quality of drinking water, damaging the soil for agriculture and causing harm to aquatic life and life in the soil [24-27]. Surveys undertaken by the European Environment Agency suggest that a considerable amount of medicines is not returned to pharmacies for proper discharge and that this is particularly the case of liquid medicines such as drops and syrups which are discharged via the sink or toilet instead [28].

European citizens are provided support for proper disposal of unused medicines: In Austria and in all further but two EU Member States patients and their families can return unused medicines to a pharmacy [29]. Additionally, medicines can also be handed over to a public waste collection point in many European countries [30]. In Vienna, the capital city of Austria, public collection points are maintained by the municipality of Vienna [31].

From these services, further information about the non-use of medicines can be gained. In several high-income countries a growing body of literature [32] is available on patients and their families returning medicines to the pharmacies, including investigations about their reasons for doing this. Death of the patient, change in prescription, too large pack sizes, repeat filling of prescriptions without assessing the amount at hand were identified as major reasons why medicines are no longer wanted and expire unused. Furthermore, patients did not see a need for continuing medication following a therapy change by the doctor or after the subjective perception of an improvement of their conditions [19,21,33-36]. Non-adherence can stem from deliberate action of the patient related to individuals’ beliefs (intentional non-adherence) and from factors outside the conscious control of the medicine user (unintentional non-adherence) [17,37].

Less is known about the amount of medicines not discarded properly. A study from Germany estimated that around 10% of sold medicines end up in household waste [38]. A few further studies, particularly from the United States, based on (telephone) interviews, suggested that disposal via the garbage, sink and toilet is common practice [25,27,39-44]. A review of existing literature concluded that measurements of the quantity of medicines discarded in proportion to total sold are not available and that the best estimates identify a range of 5% to 10% [32].

In September 2009 Austria’s leading tabloid daily newspaper stated that as much as 50% of medicines ended up in the waste [45]. However, no scientific investigation on this issue has been undertaken in Austria yet.

In response to speculations about medicines waste and concerns for policy-makers about this aspect of irrational use of medicines, we analyzed a sample of medicines selected from household garbage in Vienna with the aim of learning whether and which medicines end up unused in normal household waste.

## Methods

### Study design

We investigated as to whether and which medicines end up unused in household garbage by analyzing a sample of pharmaceutical waste drawn by the Municipal Waste Department (‘Magistratsabteilung 48’) of the City of Vienna.

We knew that the Vienna Municipal Waste Department has been performing analyses of special types of waste (e.g. recovered paper, discarded metal, used plastic) on a regular basis. Every five years medicines are collected from normal household waste in four tranches during that year.

We addressed the Vienna Municipal Waste Department in November 2009 and inquired whether after finalization of their analyses (e.g. assessing the weight in proportion to total waste) they could share a pharmaceutical waste sample with us for scientific research.

We received permission to use the pharmaceutical waste which was collected during six weeks of the last quarter of 2009. We obtained the pharmaceutical waste sample at the end of November 2009. We did a manual search of the sample, reported the key characteristics and outcome measures and did statistical analyses in April/May 2010.

### Data collection procedure

The pharmaceutical waste was collected by staff of the Vienna Municipal Waste Department. They separated from the other garbage what they considered as medicines, including packages, blisters, loose tablets, containers for ointments etc. A total of 5,000 tons of household garbage was searched for medicines; this was considered representative by the Vienna Municipal Waste Department. We are not aware about specific selection criteria, and we had no control of the design of the primary data collection.

### Study area and time-frame

The pharmaceutical waste was collected from normal household garbage in all districts of Vienna between 12 October and 24 November 2009. The sample did not include medicines delivered by hospitals or community pharmacies (special waste).

### Data source

Our data source was a bag of packages (empty or with contents), loose blisters (empty or with contents), and a few medical devices. It weighted around five kilograms.

### Outcome measures

The key outcome measures were ‘packs without contents’ and ‘packs with contents’. We understood as ‘contents’ a blister containing one or more tablets or a jar containing some ointment, for instance. Further outcome measures were the prescription status, the country of origin, the therapeutic group, the dose form and the expiry date of the packs.

### Investigation and reporting of the pharmaceutical waste sample

We manually investigated the sample of pharmaceutical waste by checking every single item and compiling a data set for further analysis. Medical devices were excluded. Our unit of measurement was packs. Individual blisters were investigated regarding their expiry date, and they were attributed to a defined pack in cases that they were of the same pharmaceutical presentation (same trade name, same pharmaceutical form, same size of the blister, same strength if indicated on the blister) and if they displayed the same expiry date.

We documented the data in an Excel® file. We reported for each pack the following information wherever available: trade name, active ingredient, strength, dose form, pharmaceutical form, pack size, information on the content, expiry date, marketing authorization holder, the country of origin and the prescription status. Furthermore, we attributed the therapeutic classification (e.g. alimentary tract and metabolism, cardiovascular) based on the ATC (anatomic, therapeutic and chemical) classification of the Oslo WHO Collaborating Centre for Drugs Statistic Methodology [46]. Information on the contents was documented in the Excel® file as follows: empty pack, loose blister (or other container) with or without tablet(s) (or other contents), pack containing blister(s) with or without tablets, and a note as to whether a full pack had ended up in garbage.

### Statistics and data analysis

We performed descriptive statistics to analyze the data (total of packs as well as the two sub-sets of packs with contents and without contents) with regard to the country of origin, prescription status, therapeutic group, dose form and expiry date. Furthermore, we tested whether these variables had impact on whether, or not, contents were left in the packages. STATA 12 was used to perform descriptive statistical analyses (cross-tabulation) and to undertake the logistic regression analyses.

## Results

We identified a total of 152 medicines packs. Table 1 provides information about the characteristics of the total of packs and the sub-groups of packs without and with contents.

**Table 1:** Analysis of the total data set and the sub-groups of packs without and with contents

**Packs**	**Total (n,%**^**a**^**)**	**Packs without contents (n,%**^**a**^**)**	**Packs with contents (n,%**^**a**^**)**
**Total**	**152 (100%)**	**130 (100%)**	**22 (100%)**
***Sub-group related to the prescription status (n = 152)***			
POM	113 (74.3%)	99 (76.2%)	14 (63.6%)
OTC	39 (25.6%)	31 (23.8%)	8 (36.3%)
***Sub-group related to the country of origin of the pack (n = 152)***			
Austria	139 (91.5%)	118 (90.8%)	21 (95.5%)
Germany	6 (4.0%)	6 (4.6%)	0 (0%)
France	1 (0.7%)	1 (0.8%)	0 (0%)
Greece	1 (0.7%)	1 (0.8%)	0 (0%)
China	1 (0.7%)	0 (0%)	1 (0.8%)
Not attributable	4 (2.6%)	4 (3.1%)	0 (0%)
***Sub-group related to the therapeutic group (n = 152)***			
A: Alimentary tract and metabolism	15 (9.9%)	14 (10.8%)	1 (4.6%)
B: Blood and blood forming organs	4 (2.6%)	4 (3.1%)	0 (0%)
C: Cardiovascular system	36 (23.7%)	31 (23.9%)	5 (22.7%)
D: Dermatologicals	2 (1.3%)	2 (1.5%)	0 (0%)
G: Genito urinary system and sex hormones	2 (1.3%)	2 (1.5%)	0 (0%)
J: Anti-infectives for systemic use	5 (3.3%)	4 (3.1%)	1 (4.5%)
M: Musculo-skeletal syst.	17 (11.2%)	13 (10.0%)	4 (18.2%)
N: Nervous system	16 (10.5%)	15 (11.5%)	1 (4.5%)
R: Respiratory system	10 (6.6%)	6 (4.6%)	4 (18.2%)
Other ATC code or not attributable	45 (29.6)	39 (30.0%)	6 (27.2%)
***Sub-group related to the dose form (n = 152)***			
Oral	132 (86.8%)	119 (91.5%)	13 (59.1%)
Parental	6 (4.0%)	5 (3.8%)	1 (4.6%)
Nasal	1 (0.7%)	0 (0%)	1 (4.6%)
Pulmonary	1 (0.7%)	1 (0.8%)	0 (0%)
Dermal	10 (6.7%)	5 (3.7%)	5 (22.7%)
Eye	1 (0.7%)	0 (0%)	1 (4.6%)
Dental	1 (0.7%)	0 (0%)	1 (4.6%)
***Sub-group related to the expiry status and expiry dates (n = 136)***^b^			
*Expiry status*			
Expired	7 (5.2%)	3 (2.6%)	4 (19.0%)
Non expired	129 (94.8%)	112 (97.5%)	17 (81.0%)
*Expiry dates*			
1999	1 (0.7%)	0 (0%)	1 (4.8%)
2002	1 (0.7%)	0 (0%)	1 (4.8%)
2007	1 (0.7%)	1 (0.9%)	0 (0%)
2008	1 (0.7%)	0 (0%)	1 (4.8%)
1/2009 – 9/2009^c^	3 (2.2%)	2 (1.7%)	1 (4.8%)
12/2009^c^	1 (0.7%)	0 (0.9%)	1 (0%)
2010	21 (15.4%)	18 (15.7%)	3 (14.3%)
2011	31 (22.8%)	26 (22.6%)	5 (23.8%)
2012	43 (31.6%)	37 (32.2%)	6 (28.6%)
2013	24 17.6%)	21 (18.3%)	3 (14.3%)
2014	9 (6.6%)	9 (7.8%)	0 (0%)

^a^Total does not always sum to 100% because of rounding errors ^b^No expiry date could be identified for 16 packs ^c^Collection date was October/November 2009.

### Total of packs

74% of the packs were prescription-only medicines (POM) and 26% OTC (Over-the-Counter) medicines. 139 packs (92% of the total) were packs marketed in Austria. Nine packs were identified as being from abroad: they were mainly from Germany (6), one from France and Greece respectively, and one pack had a Chinese label. Cardiovascular medicines accounted for the highest share (24%). Around 10% of packs were from medicines for the musculo-skeletal system, the nervous system and for the alimentary tract and metabolism respectively. Most packs were oral medicines (87%), usually solid oral; whereas dermal use (7%) and parental use (4%) had lower shares. 5% had expired before the date of collection (October/November 2009), 95% had not expired. Expiry dates were up till the year 2014; the highest shares of medicines were due to expire in 2011 (23% of the packs for which we could identify the expiry date) and 2012 (32%).

### Packs with contents

22 packs (14.5% of the total) contained contents. The range of contents left in the packs varied: In several packages 2, 3 or 6 tablets were left. In total we found 3 full packs. With regard to liquids and ointments, the sample included bottles of one sixth, one third and two thirds full, a container full of crème and two others with one quarter and one half of their contents. The results for the small sub-set of packs with contents were different compared to the full sample: The share of OTC medicines (36%) was higher. Medicines of the respiratory system, e.g. to treat a cold, and medicines of the musculo-skeletal system, particularly anti-flammatory medicines, accounted for higher shares within the group of packs with contents (18% in both cases) in comparison to the total (11% and 7% respectively). Within the sub-group of packs with contents, the oral form was the most frequently identified dose form, nonetheless the share of oral medicines was lower (59% compared to 87% in total of packs). Medicines for dermal use were more frequently found in this sub-group (23% compared to 7% in total). The share of packs which had expired was nearly 4 times (19%) the packs with contents compared to the full sample (5%). Two packs discarded had expiry dates of around a decade ago (1999 and 2002), and both contained contents.

We tested the assumption as to whether the prescription status, the therapeutic group, the dose form and the expiry date influence the disposal of packs with some original content in household waste. No statistically significant association was found except for the expiry date. The probability of a pack with contents ending up in household waste is 0.7 times compared to an expiry date of one year earlier [OR = 0.69 (95%CI 0.51:0.92), p = 0.014].

## Discussion

The major result of our study is that some medicines end up unused, or partially unused, in household garbage. In total, 14.5% of the packs had contents. These findings challenge the high figures, as of 50%, circulated in the tabloid press [45], which caused concerns with policy-makers and the public.

Our study analyzed for the first time in Austria the contents of a pharmaceutical waste sample and thus provides evidence to an under-researched area. While from other countries studies on medicines returned to pharmacies, frequently supplemented by a investigation exploring the reasons for the non-use of the medicines [18-23,33-35] as well as surveys based on interviews about disposal practices of households [25-27,39-41,43,44,47] are available, to the best knowledge of the authors no such investigation has ever been undertaken in Austria.

However, our study investigated normal household waste and did not survey how many medicines were improperly discarded of via other channels, in particular liquid medicines such as drops and syrups via the sink or the toilet. In Germany, 43% of households questioned admitted to having thrown liquids into the sink or toilet at least occasionally, compared to 16% of the surveyed people doing this with single tablets [41]. A survey from the United States showed even higher shares: More than half of the people interviewed reported storing unused and expired medications in their homes, and more than half had flushed them down a toilet [47]. These and further findings from a literature review on this topic [40] suggest that in the case of improper discharge solid medicines are more likely to be discarded in normal household garbage whereas liquids are disposed of via toilets and sinks. In our sample we had very few medicines in liquid forms, whereas by far the highest percentage of our sample (in total and in packs with contents) was medicines for solid oral use. This could be understood as an indication that people in Vienna also have this two-tiered approach to improperly discharge medicines: solids in the household garbage and liquids into sinks and toilets. In our sample particularly medicines for dermal use (e.g. crème, ointments) ended up unused or partially used in waste (half of all dermal medicines had contents).

The legal framework and information policies are likely to impact medicines disposal behaviour [36,48]. In Austria, unused medicines are required to be returned to pharmacies, or to public collection points, which are, for instance, offered by the municipality of Vienna [28-30]. However, there are no sanctions for wrongful disposal; the focus is put on information policies by the Vienna Municipal Waste Department [31,49] and pharmacies. No information is available for Austria whether people believe that some forms of improper use would be correct. From a study performed in the United States, for instance, it is known that more than 35% of the respondents considered it acceptable to flush down medicines the toilet, and more than 21% believed it acceptable to rinse them down the sink [47].

Cardiovascular medicines, medicines of the musculo-skeletal system, the nervous system and for the alimentary tract and metabolism were the major therapeutic groups (in packs without and with contents). Medicines of the respiratory system were particularly likely to end up unused or partially used in the household garbage. These therapeutic groups account for high consumption among reimbursed medicines in Austria [50] and are among the medicines most frequently returned to pharmacies, as evidence from other countries shows [21-23,33,51,52]. The rather high share (40%) of medicines for treatment of the respiratory system with contents suggests that patients purchase medicines for self-medication to treat minor ailments such as a cold with the intention to use them for a defined period of time, probably until some improvement or recovery will be achieved, and afterwards they will discard the medicines with contents in household garbage. This assumption is supported by the, however not statistically significant, fact that in the group of OTC medicines a higher share of packs (21%) had contents compared to the group of prescription-only medicines (12%) and the full data set (15%). Adherence appears to be higher when medicines are prescribed by a doctor [25].

A Swiss study according to which one third of all packages disposed were not even opened [16] suggested that patients did not start the medication at all. The results of our study did not confirm this pattern: we identified only very few packs which were completely full. Our findings, which suggest that patients were apparently committed to start the therapy but then stopped it, are supported by an opinion poll performed in Austria which stated that more than 50% of the people questioned reported to have left only a few tablets in the pack [53].

According to this Austrian opinion poll [53] the two major reasons indicated why patients did not fully consume the medicines were the subjective feeling of an improvement of their well-being (52%) and too large pack sizes (25%), followed by the advice of the doctor to stop therapy, a negative feeling towards too much medication, fears about adverse reactions, forgetting to take the medicines and stopping of medication after having read the patients’ leaflet. Similar reasons were reported in the surveys with patients returning medicines to pharmacies in other high-income countries [14,19,32-35].

The non-use of prescribed medicines might be incentivized by the lack of price sensitivity of Austrian patients. Prices of medicines reimbursed by the Social Health Insurance are not indicated on the packs, and the pharmacy retail prices are not published. This lack of knowledge about prices is also attributable to the fact that patients in Austria do not have to co-pay apart from a fee (€ 4.80 in 2009) per prescribed item [54]. Patients in other European countries are likely to be more price sensitive due to a reimbursement system which asks for a percentage co-payment depending on the price of the medicine [55,56].

The possible market entry of counterfeits is increasingly causing concern in European countries [57,58]. Till now, the official distribution chain in Austria has not been targeted by counterfeits, but the risk exists when patients purchase medicines from internet pharmacies abroad (internet pharmacies located in Austria are currently not allowed [59]). The waste sample which we analyzed did not suggest a penetration of the Austrian market with counterfeits. Less than 10% of the packs of the sample were not Austrian, in most cases their origin could be identified from other European countries, mainly Germany. Since the medicines packs from other European countries contained no contents, we assume that tourists brought these medicines with them and used them up in Vienna.

This study has some limitations. The methodological approach for drawing the pharmaceutical waste sample was decided by the Vienna Waste Department whose staff, without specific pharmacological training, did the first collection of the waste sample. But we believe that the qualification bias did in general not distort the results because the number of non-pharmaceuticals included was low. We received a bag of different items, containing half-full and empty packages and loose blisters, and we had to base our analysis on some assumptions. For retracing loose blisters to a pack, for instance, we attributed the blisters of a pharmaceutical presentation which had the same expiry date to one pack which might have resulted in an under-estimation of the number of packs, and thus a possible over-estimation of the share of packs with contents. Potentially cofounding variables were not statistically significant, which is likely to be attributable to the small size of the sample, and particularly of the sub-groups. But it should be recalled that the comparably small sample size resulted from the Municipal Waste Department’s representative collection in all 23 Viennese districts during a reasonable time-period of six weeks. We did not have comparative data in order to address possible seasonal variation.

Notwithstanding these limitations, the study adds information to an area of scant knowledge. For the first time in Austria, we have some evidence-based indication of the extent and types of medicines discarded in household garbage. The findings call for further action: First, patients’ adherence to medicines requires to be improved. We believe that community pharmacists, in their role as health professionals with tasks far beyond ‘medicine sellers’ [60-64], can play a major role via their communication and counseling activities to support public actions and campaigns to improve adherence to medicines. Second, public authorities and health professionals are called upon to inform about proper discharge in order to prevent ill-feared consequences for the environment. There is evidence that patients informed by pharmacists or other health professionals are more likely to discharge appropriately [27,40,44,47]. Finally, the safety aspect is another challenge to be addressed: one medicine in our sample (Chinese label) was apparently bought over the internet without consultation.

Our investigation of a pharmaceutical waste sample drawn in Vienna provides insight into one specific aspect of non-use and waste of medicines. It is a starting point. But we continue to lack information about the extent of medicines improperly discharged via other channels, and we have scant knowledge of the amount of medicines, expired and/or not intended to be taken, stored in Viennese, and Austrian, households. Further research is required: We recommend performing a study of medicines returned to pharmacies, if possible accompanied by a survey about the reasons for the non-use. Pharmaceutical waste analyses in other Austrian provinces and a follow-up investigation of another Viennese waste sample in future are suggested. Any study which helps understanding the reasons of patients’ non-adherence is welcome. Findings about the quantitative aspects of medicines wasted should be linked to financial parameters (cost of medicines, including those borne by public payers) in order to assess the dimensions of wasted resources.

## Conclusions

The study proved that some medicines ended up unused or partially used in normal household garbage in Vienna. In total, 14.5% of the medicines packs of our sample had contents. Our results did not confirm speculations circulated in the public debate about a high percentage of unused medicines improperly discarded but it is an indication for irrational use of medicines. More research is needed, particularly on the reasons why patients stop a therapy and which measures are most appropriate to improve patients’ adherence. In the meantime, pharmacists are encouraged to continue playing a major role in the communication and counseling of patients in order to promote a more rational use of medicines and a proper discharge of medicines.

## Competing interest

No financial and non-financial competing interests.

## Authors’ contributions

The study was performed in the Health Economics Department (meanwhile designated a WHO Collaborating Centre) at the Austrian Health Institute (Gesundheit Österreich GmbH). Sabine Vogler, Christine Leopold and Claudia Habl are staff members of the Austrian Health Institute. Christel Zuidberg was a Pharmacy Master’s student at Utrecht University who did an internship at the Austrian Health Institute at the time of the research. SV, CL, CZ and CH were equally involved in the design of the study. CH approached the Vienna Waste Department to obtain the pharmaceutical waste sample. CZ, supported by CL, did the manual investigation of the items of the sample and compiled the results. CZ and CL drafted the first version of this paper. SV did the literature search and wrote further versions of the manuscript, including the one submitted. All authors have read the final version of this article and approved it. All authors have read the final version of the article and approved it. All authors are aware of the submission and agree with it.

## Author’s information

Christel Zuidberg: Currently working in community pharmacy in the Netherlands, at the time of the research Pharmacy Master’s student at the WHO Collaborating Centre for Pharmaceutical Policy and Regulation, Division of Pharmacoepidemiology and Clinical Pharmacology, Utrecht Institute for Pharmaceutical Sciences, and intern at the Health Economics Department, Gesundheit Österreich GmbH/Geschäftsbereich ÖBIG – Austrian Health Institute.
